# *miR-140-5p* attenuates chemotherapeutic drug-induced cell death by regulating autophagy through inositol 1,4,5-trisphosphate kinase 2 (IP3k2) in human osteosarcoma cells

**DOI:** 10.1042/BSR20160238

**Published:** 2016-10-14

**Authors:** Renxiong Wei, Gang Cao, Zhouming Deng, Jiajia Su, Lin Cai

**Affiliations:** * Department of Orthopaedics, Zhongnan Hospital of Wuhan University, Wuhan 430071, China; † Department of Neurosurgery, Taihe Hospital, Hubei University of Medicine, Shiyan 442000, China; ‡ Department of Radiology, Hubei Cancer Hospital, Wuhan 430079, China

**Keywords:** autophagy, drug resistance, miRNA-140, osteosarcoma cells, 1, 4, 5-trisphosphate (IP3) signalling

## Abstract

Acquisition of drug-resistant phenotypes is often associated with chemotherapy in osteosarcoma. A number of studies have demonstrated a critical role for autophagy in osteosarcoma development, therapy and drug resistance. However, the molecular mechanisms underlying the autophagy-mediated chemotherapy resistance of osteosarcoma cells remain largely unknown. In the present study, we determined the autophagy and microRNA-140 (*miR-140-5p*, miRBase ID: MIMAT0000431) expression induced by chemotherapeutic drugs in osteosarcoma cells. Then we determined the promotory role of *miR-140-5p* to the chemotherapy-induced autophagy. Our results demonstrated that *miR-140-5p* expression was highly induced during chemotherapy of osteosarcoma cells, and this was accompanied by up-regulated autophagy. The increased *miR-140-5p* expression levels up-regulated anticancer drug-induced autophagy in osteosarcoma cells and ameliorated the anticancer drug-induced cell proliferation and viability decrease. Importantly, *miR-140-5p* regulates this context-specific autophagy through its target, inositol 1,4,5-trisphosphate kinase 2 (IP3k2). Therefore, the results of the present study demonstrated that *miR-140-5p* mediated drug-resistance in osteosarcoma cells by inducing autophagy. The present study provides evidence of miRNA regulation of autophagy through modulation of IP3 signalling. The present study recognized a novel mechanism of chemoresistance in osteosarcoma cancers.

## INTRODUCTION

Osteosarcoma is the eighth most common type of cancer found in children and adolescents, accounting for 2.4% of all malignancies in paediatric patients and ∼20% of all primary bone cancers [1]. Chemotherapy is the first choice treatment for osteosarcoma, with multiple anticancer drugs, including doxorubicin (Dox), cisplatin (Cis) and high-dose methotrexate [2,3]. In the last three decades, neoadjuvant chemotherapy has increased the long-term survival rate of osteosarcoma patients from <20 to ∼80% [4–6]. However, patients that are less responsive to these drugs have a poor prognosis. In addition, the frequent acquisition of drug resistance and the occurrence of ‘secondary malignancies’ are often associated with chemotherapy and are significant obstacles to achieving favourable outcomes. Thus, it is important to identify the molecular mechanisms underlying the drug resistance of osteosarcoma cancer cells.

Autophagy is a universal process whereby cellular components and damaged organelles are sequestered within autophagosomes for lysosomal degradation. Autophagy has proven to be an essential pathway for cellular homoeostasis. In addition to removing dysfunctional proteins and organelles, autophagy provides amino acids, monosaccharides, nucleic acids and lipids during times of nutrient deprivation [7,8]. Autophagy is a key pathway for cell survival but, if protein loss becomes excessive, cell death will result. This degradative pathway has been implicated in the progression of a number of diseased states including cancer. Suppressed autophagy can result in net protein gain and neoplastic growth, and defects in autophagy have been implicated in poor outcomes for hepatocellular carcinoma [9]. To the contrary, autophagy promotes cell survival in tumours undergoing nutrient deprivation or chemotherapy. The overproduction of the autophagy protein, LC3B (microtubule-associated protein 1 light chain 3B), is associated with tumour growth and poor prognosis in aggressive pancreatic, colorectal and breast carcinoma [10–12].

During tumour development, autophagy is enhanced to promote cell survival under ischaemic conditions [13–15]. Autophagy can also enhance cell survival by removing organelles damaged by chemotherapy agents [16,13,17]. Resistance of osteosarcoma cell lines to Dox, Cis and methotrexate has been shown to be due to the induction of autophagy by the DNA-binding protein HMGB1 (high mobility group box 1) [18]. On the other hand, autophagy is one of three primary venues of cell death, which also includes apoptosis and necrosis. Many existing chemotherapy drugs act by inducing apoptosis whereas others promote autophagy-mediated cell death of neoplastic cells [19,20]. Given that autophagy can promote cell survival or cell death, its regulation is critical for the developing tumour.

There are two primary regulatory pathways of autophagy: MTOR (mechanistic target of rapamycin), a negative regulator, and PtdIns3K (class III phosphatidylinositol 3-kinase), a positive regulator. MTOR inhibits the ULK1/2 (mammalian orthologues of yeast Atg1) complex, which activates autophagy by stimulating PtdIns3K activity [21]. The MTOR inhibitor, rapamycin, induces autophagy-mediated cell death in glioma cells [22]. PtdIns3K synthesizes phosphatidylinositol 3-phosphate, which provides a docking site for ATG proteins at the sequestering membranes of the forming autophagosome [23,24]. Chemoresistance is attenuated in hepatocarcinoma cells when treated with the PtdIns3K antagonist, 3MA (3-methyladenine) [25]. Both pathways modulate the lipidation of LC3B by presumably regulating the activities of ATG4, ATG7 or ATG3. Of the four autophagins (ATG4A, ATG4B, ATG4C and ATG4D) identified, Yin and co-workers have shown that ATG4B had the highest catalytic efficiency for cleaving the C terminus of LC3B [26]. Once the C-terminal glycine of LC3B is exposed by ATG4B, ATG7 in an ATP-dependent manner activates LC3B for delivery to ATG3, which conjugates LC3B to phosphatidylethanolamine. The lipidation of LC3B anchors this protein to the forming autophagosome where it promotes membrane expansion to enlarge the autophagosome thus increasing the amplitude of autophagy [27]. The lipidated LC3B is either degraded within the autolysosome or cleaved by ATG4B and the LC3B recycled. ATG4B provides the cell with enough LC3B to amplify autophagy and recycles the lipidated LC3B to sustain autophagy [28].

MicroRNAs (miRNAs) are family of endogenous non-coding RNA molecules that comprise 22 nucleotides, which regulate gene expression [29] in organisms ranging between nematodes and humans and in a broad array of mammalian cell processes [30]. Recently, miRNAs have been associated with cell chemosensitivity or chemotherapy resistance in a variety of cancer cell types [31], including osteosarcoma [32]. *miR-140* was reported to be involved in the chemoresistance of osteosarcoma cells via the suppression of histone deacetylase [4], which in turn reduced cell proliferation [32]. Furthermore, an increasing number of studies have demonstrated that miRNA molecules regulate cellular autophagy processes [33–35]. Zhu et al. [34] reported that *miR-30a* targets *beclin 1*, resulting in decreased autophagic activity. In addition, Brest et al. [35] showed that a *miR-196*-based alteration in the expression of immunity-related GTPase family M protein can affect the efficacy of autophagy. However, the role of miRNAs in autophagy-mediated chemotherapy resistance in osteosarcoma remains unknown.

In the present study, we determined the targeting role of *miR-140-5p* (miRBase ID: MIMAT0000431) to inositol 1,4,5-trisphosphate kinase 2 (IP3K2), the regulation of *miR-140-5p* on the IP3K2-mediated cell autophagy during chemotherapy, and the suppression of *miR-140-5p* inhibitor in the cell proliferation of osteosarcoma cells. Thus, we identified the tumour suppressive role of *miR-140-5p* inhibitor in osteosarcoma cells *in vitro*.

## MATERIALS AND METHODS

### Cell culture and reagents

Human osteosarcoma cell lines (Saos-2 and MG-63) were obtained from the Cell Resource Center of the Chinese Academy of Medical Sciences. The cells were cultured in Eagle's Minimum Essential Medium (Invitrogen) or McCoy's 5A Modified Medium (Invitrogen) supplemented with 10% FBS (GIBCO), and were incubated at 37°C with 5% CO_2_. Antibodies against GAPDH, LC3-II and p62 were obtained from Santa Cruz Biotechnology and rapamycin was purchased from Sigma–Aldrich. The coding sequence of microtubule-associated protein 1-LC3 fusion with GFP was synthesized and cloned into pcDNA3.1(+) to construct the LC3-GFP-expressing plasmid.

### Cell transfection

*miR-140-5p* mimic, *miR-140-5p* inhibitor and the corresponding control oligonucleotides (purchased from RiboBio) were transfected into cells as described previously [36]. The sequence of *miR-140-5p* mimics was 5′-UGAGAACUGAAUUCCAUGGGUU-3′, and miR-control was 5′-UUC UCC GAA CGU GUC ACG UTT-3′. The sequence of *miR-140-5p* inhibitor was 5′-AA CCC AUG GAA UUC AGU UCU CA-3′, and miR-NC was 5′-UCU ACU CUU UCU AGG AGG UUG UGA-3′. siRNAs targeting IP3K2 were obtained from RiboBio and sequences were 5′-GCU AUC AAC UGC AGA GAU U-3′. The IP3K2 siRNA and control siRNA transfections were conducted as recommended by the manufacturer.

Quantitative GFP-LC3 light microscopy autophagy assays were performed in Saos-2 cells with various treatments. Cells were grown to 80% confluency and were transfected with a GFP-LC3-expressing plasmid using Lipofectamine 2000 (Invitrogen Life Technologies). At 24 h following transfection, the cells were subjected to 0.2 μg/ml Dox (Sigma–Aldrich) or 20 μM Cis (Sigma–Aldrich) for an additional 24 h. In a separate experiment, cells were simultaneously and additionally transfected with 20 nM *miR-140-5p* and analysed with fluorescence microscopy. The number of punctate GFP-LC3 dots in each cell was counted and at least 100 cells were included for each group.

### miRNA extraction and quantitative PCR

Total miRNA extraction was performed using a mirVana miRNA Isolation kit (Ambion). Quantification of *miR-140-5p* expression was conducted using the mirVana qRT-PCR miRNA Detection kit (Ambion), where U6 small nuclear RNA was used as an internal control, according to the protocol previously described [37]. The specific primer of *miR-140-5p* was: GTC GTA TCC AGT GCA GGG TCC GAG GTA TTC GCA CTG GAT ACG ACC TAC CAT.

For mRNA detection, total RNA was extracted using TRIzol reagent (Life Technologies), according to the manufacture's instruction. The mRNA expression was determined by using the standard SYBR-Green RT-PCR kit (Takara), in accordance with the manufacturer's instructions. The specific primers were as follows: IP3K2, 5′-TTA CTC AAG GAC GCG GTC TGT GAT C-3′ (forward) and 5′-ATT GGC CCC AGC TTG CTT-3′ (reverse). GAPDH was used as an internal control with primers: 5′-AGC CTT CTC CAT GGT GGT GAA-3′ (forward) and 5′-ATC ACC ATC TTC CAG GAG CGA-3′ (reverse).

### Western blot analysis

Cell extracts were prepared according to the standard protocol, and protein expression levels were detected by western blot analysis using polyclonal (rabbit) anti-LC3-II, anti-p62 or anti-GAPDH antibodies. Goat anti-mouse IgG or goat anti-rabbit IgG (Pierce Biotechnology) secondary antibodies, that were conjugated to horseradish peroxidase, were used for detection via an enhanced chemiluminescence detection system (Super Signal West Femto, Pierce Biotechnology).

### Cell proliferation assay

Cell viability was expressed as the relative percentage of viable cells to control human umbilical vein endothelial cells. For the proliferation assay, following transfection with *miR-140-5p* mimics or miRNA control, cells were incubated with Cell Counting Kit-8 (CCK-8; Dojindo Molecular Technologies). The absorbance of each well at 450 nm was detected following visual colour occurrence at 24, 48 or 72 h. Independent experiments were performed in triplicate.

### Ca^2+^ measurements

Fura-2 fluorescence was utilized to determine intracellular Ca^2+^ concentrations [38]. Cells were loaded with Fura-2/AM (2 μM, Invitrogen) for 20 min at 37°C. Cells were excited alternatively at 340 and 380 nm through an objective (Fluor 40×/1.30 oil) built in an inverted phase-contrast microscope (Axiovert100, Zeiss). Emitted fluorescence intensity was recorded at 505 nm. Data were acquired using specialized computer software (Metafluor, Universal Imaging). Cytosolic Ca^2+^ activity was estimated from the 340 nm/380 nm ratio. Store-operated Ca^2+^ entry (SOCE) was determined by extracellular Ca^2+^ removal and subsequent Ca^2+^ readdition in the presence of thapsigargin (1 μM, Invitrogen). For quantification of Ca^2+^ entry, the slope (delta ratio/s) and peak (delta ratio) were calculated following readdition of Ca^2+^. Experiments were performed with Ringer solution containing: 125 mM NaCl, 5 mM KCl, 1.2 mM MgSO_4_, 2 mM CaCl_2_, 2 mM Na_2_HPO_4_, 32 mM HEPES, 5 mM glucose, pH 7.4. To reach nominally Ca^2+^-free conditions, experiments were performed using Ca^2+^-free Ringer solution containing: 125 mM NaCl, 5 mM KCl, 1.2 mM MgSO_4_, 2 mM Na_2_HPO_4_, 32 mM HEPES, 0.5 mM EGTA, 5 mM glucose, pH 7.4.

### Statistical analysis

For GFP-LC3 dot number analysis, relative *miR-140-5p* expression, conversion of LC3-I to LC3-II, relative expression of p62 against GAPDH and CCK-8 measurements, the statistical evaluations are presented as the mean ± S.E. Data were analysed using the Student's *t* test. All data were analysed by the SPSS v16.0 (SPSS). *P*<0.05 was considered to indicate a statistically significant result.

## RESULTS

### *miR-140-5p* expression increases in osteosarcoma cells following treatment with chemotherapy agents

**Figure 1:**
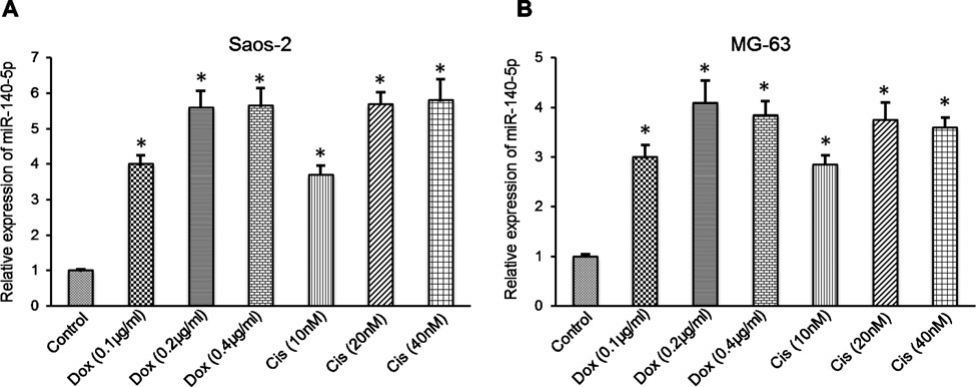
*miR-140-5p* expression was up-regulated in osteosarcoma cells following treatment with chemotherapeutic drugs qPCR analysis showing the relative *miR-140-5p* expression to U6 in (**A**) Saos-2 and (**B**) MG-63 cells. All the experiments were performed in triplicate. *P<0.05 compared with control.

The role of miRNAs in chemotherapy-induced autophagy of cancer cells remains unknown. To screen possible miRNAs that may be important for anticancer drug-induced autophagy in osteosarcoma cells, miRNA expression levels were analysed by microarray in osteosarcoma cells following treatment with Dox. *miR-140-5p* was demonstrated to be the most highly-expressed miRNA. Thus, the expression level of *miR-140-5p* was quantified in Saos-2 and MG-63 cells following treatment with Dox or Cis. The results indicated that treatment with 0.2 μg/ml Dox or 20 μM Cis significantly up-regulated the *miR-140-5p* expression levels in the two cell lines. A quantitative PCR (qPCR) assay demonstrated that significantly higher expression levels of *miR-140-5p* were induced in Saos-2 or MG-63 cells following Dox or Cis treatment (Figures 1A and 1B). Therefore, *miR-140-5p* expression is induced *in vitro* during anticancer drug therapy in osteosarcoma cells.

### Inhibition of *miR-140-5p* promotes the anticancer drug-induced cell proliferation decrease

**Figure 2:**
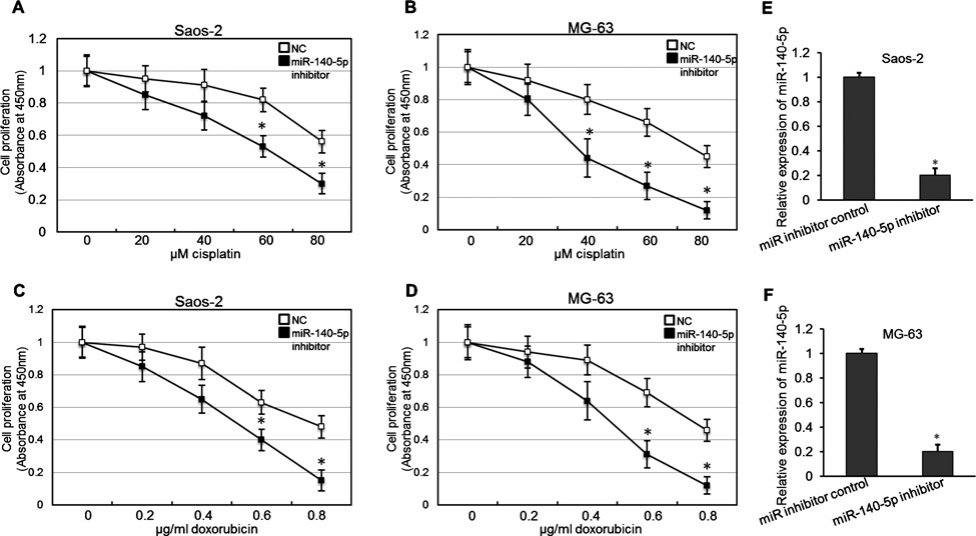
Inhibition of *miR-140-5p* ameliorated the anticancer drug-induced cell proliferation decrease *in vitro* (**A** and **B**) Osteosarcoma cells were transfected with *miR-140-5p* inhibitor or a scrambled control. Forty-eight hours after transfection, Cis was added and after 24 h cell viability was determined with a CCK-8 assay. Independent experiments were performed in triplicate. *P<0.05 compared with control. (**C** and **D**) Growth curves showing the cell proliferation following Dox treatment and *miR-140-5p* inhibitor or miR-control transfection in osteosarcoma cells. *P<0.05 compared with control. (**E** and **F**) When transfected with *miR-140-5p* inhibitor or miR inhibitor control, real-time PCR was used to validate the changes of *miR-140-5p* expression in Soas-2 and MG-63 cells. *P<0.05.

To determine the possible effect of *miR-140-5p* on osteosarcoma cell proliferation, the proliferation of Saos-2 or MG-63 cells that had been treated with Dox or Cis and transfected with *miR-140-5p* inhibitors was determined using a CCK-8 assay. As shown in Figures 2(A) and 2(B), transfection with *miR-140-5p* inhibitor gave rise to a marked increase in sensitivity after treatment with 60 and 80 μM Cis in the Saos-2 and MG-63 cell lines. As shown in Figures 2(C) and 2(D), *miR-140-5p* in transfection resulted in a dose-dependent amelioration of 0.6 and 0.8 μg/ml Dox-induced cell proliferation inhibition in the Saos-2 and MG-63 cell lines. The effect of inhibiting was determined by real-time PCR shown in Figures 2(E) and 2(F). Thus, inhibition of *miR-140-5p* ameliorated the anticancer drug-induced cell proliferation decrease in osteosarcoma cells.

### Overexpression of *miR-140-5p* up-regulates anticancer drug-induced autophagy in osteosarcoma cells

**Figure 3:**
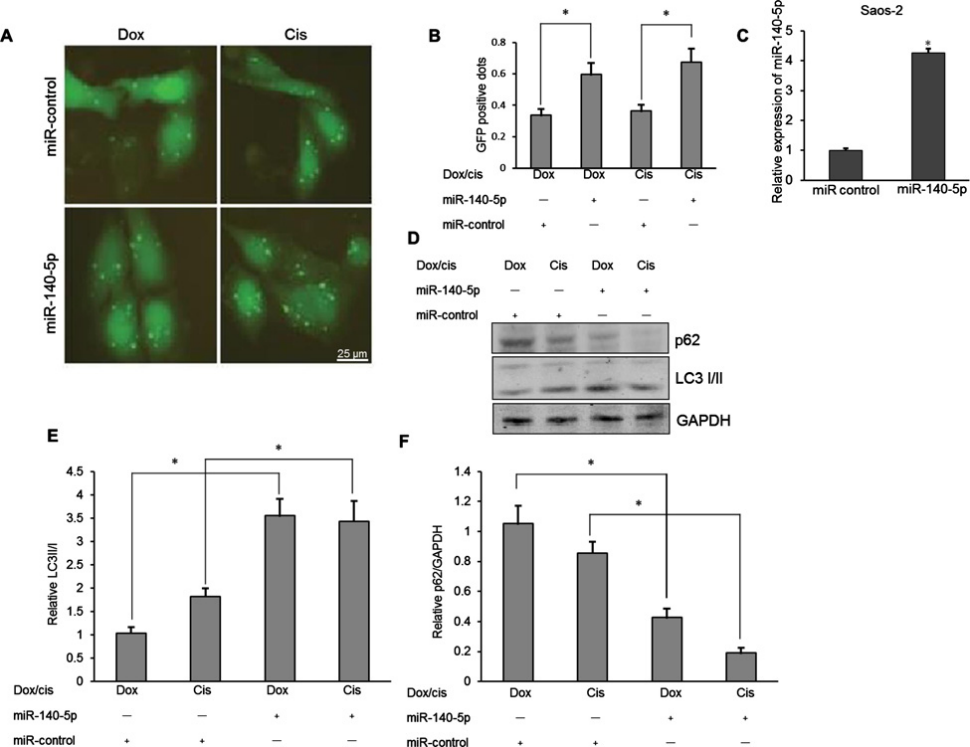
Overexpression of *miR-140-5p* up-regulated anticancer drug-induced autophagy in osteosarcoma cells (**A**) LC3 punctas under fluorescence microscopy in Saos-2 cells following 0.1 μg/ml Dox or 10 μM Cis treatment and *miR-140-5p* mimics or miR-control transfection. (**B**) Quantitative analysis of the punctate GFP-LC3 dots. (**C**) Real-time PCR was used to validate the changes of *miR-140-5p* expression after *miR-140-5p* mimics transfection. *P<0.05. (**D**) Western blotting of LC3-I/II and p62 in *miR-140-5p* mimics or miR-control-transfected Saos-2 cells. (**E**) Relative expression of LC3-II to LC3-I in *miR-140-5p* mimics or miR-control-transfected Saos-2 cells. (**F**) Relative expression of p62 to GAPDH in *miR-140-5p* mimics or miR-control-transfected Saos-2 cells. Independent experiments were performed in triplicate. *P<0.05 compared with control.

To determine the possible contribution of *miR-140-5p* to autophagy in drug-treated osteosarcoma cells, *miR-140-5p* expression was manipulated in Saos-2 cells via transfection with *miR-140-5p* mimics or miRNA control. The level of autophagy was determined in Saos-2 cells following *miR-140-5p* mimics transfection. As shown in Figures 3(A) and 3(B), there were more GFP-positive dots (LC3 punctas) in the Saos-2 cells that had been transfected with *miR-140-5p* mimics when compared with transfection with miRNA control (*P*<0.05). The effect of *miR-140-5p* overexpression was determined by real-time PCR shown in Figure 3(C). In addition, significantly higher conversion levels of LC3-I to LC3-II and decreased expression levels of p62 were also confirmed in the osteosarcoma cells transfected with *miR-140-5p* mimics (*P*<0.05 respectively; Figures 3D–3F). These results confirm that overexpression of *miR-140* contributes to anticancer drug-induced autophagy in osteosarcoma cells.

### *miR-140-5p* targets the IP3k2 protein to regulate autophagy during osteosarcoma cell death

**Figure 4:**
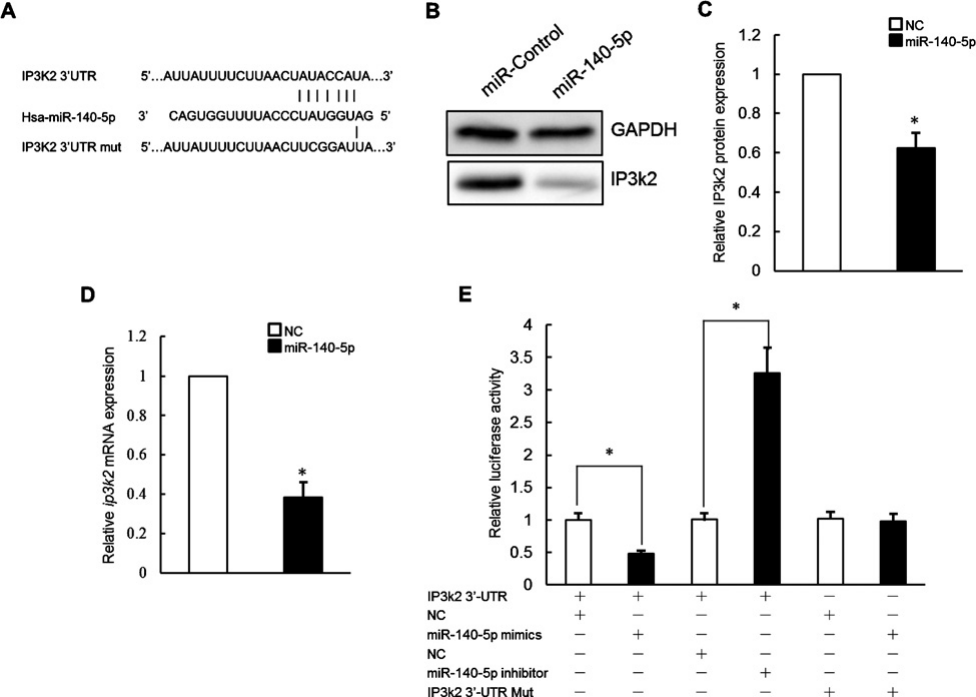
*miR-140-5p* inhibited IP3k2 expression in osteosarcoma cells (**A**) The binding sites and the corresponding mutated sequences within the *ip3k2* 3′-UTR for *miR-140-5p* were presented. (**B** and **C**) Immunoblot analyses of the effect of transient transfection of *miR-140-5p* mimics on IP3k2 protein levels in Saos-2 cells. (**D**) Quantitative RT-PCR analyses of the effect of transient transfection of *miR-140-5p* mimics on *ip3k2* mRNA levels in Saos-2 cells. (**E**) The effects of *miR-140-5p* mimics or *miR-140-5p* inhibitor on the activity of *ip3k2* 3′-UTR or *ip3k2* 3′-UTR-Mut in transiently co-transfected Saos-2 cells. The reporter activities were determined at 48 h after transfection. Data are presented as the means ± S.E.M. from *n*=3 replicates. *P<0.05.

Our next goal was to identify targets of *miR-140-5p* that influence autophagy. We used the miRNA binding site prediction programmes Pictar and Targetscan to identify candidate *miR-140-5p* target genes. Of the 31 genes, IP3k2 is involved in autophagy regulation. To investigate if *ip3k2* 3′-UTR sequences can mediate regulation by *miR-140-5p*, we examined whether *miR-140-5p* directly reduces endogenous IP3k2 levels. Saos-2 cells were transfected with either miR-control or *miR-140-5p* mimics. IP3k2 protein levels were then directly assayed by western blot. Forty-eight hours after transfection, IP3k2 protein levels were decreased significantly following transfection of *miR-140-5p* mimics as compared with negative control (Figures 4B and 4C). In a separate experiment, *ip3k2* mRNA levels were directly assayed by real time PCR after *miR-140-5p* mimics transfection. Forty-eight hours after transfection, *ip3k2* mRNA levels were decreased significantly following transfection (Figure 4D). Taken together, these results suggested that exogenous *miR-140-5p* likely inhibits IP3k2 expression via mRNA destabilization.

### *miR-140-5p* inhibits IP3k2 expression via predicted 3′-UTR target sites

To validate the functionality of the putative *miR-140-5p*/*ip3k2* 3′-UTR interaction, a reporter construct was prepared containing the full-length *ip3k2* 3′-UTR. This reporter was generated by PCR-amplifying the *ip3k2* 3′-UTR from human genomic DNA and inserting the amplicon downstream of a *Renilla* luciferase CDS. A separate firefly luciferase CDS under independent transcriptional control was also present in this construct to serve as an internal control. Co-transfection of the reporter construct along with *miR-140-5p* mimics in Saos-2 cells resulted in significantly reduced *Renilla* activity relative to co-transfection with negative control mimic or transfection of reporter construct alone (53% of negative control mimics) suggesting an inhibitory regulatory interaction between *miR-140-5p* and the *ip3k2* 3′-UTR.

To confirm that the inhibitory effect of *miR-140-5p* on *ip3k2* 3′-UTR reporter expression was mediated specifically via predicted *miR-140-5p* target sites located in the *ip3k2* 3′-UTR, mutations were introduced in the seed sequences of both target sites in the reporter construct (Figure 4A). Perfect complementarity at the seed sequence is critical for functional miRNA interactions, and mutation at this position should eliminate effective interaction between miRNA and target site. These mutant reporter constructs were then co-transfected along with *miR-140-5p* mimic into Saos-2 cells and reporter expression compared with wild-type reporter (Figure 4E). Mutation of target site partially eliminated the inhibitory effect of *miR-140-5p* mimics on reporter expression (Figure 4E). Therefore, *miR-140-5p* mediates its inhibitory effect on *ip3k2* 3′-UTR reporter expression by interacting with at least one of predicted target sites in the *ip3k2* 3′-UTR.

**Figure 5:**
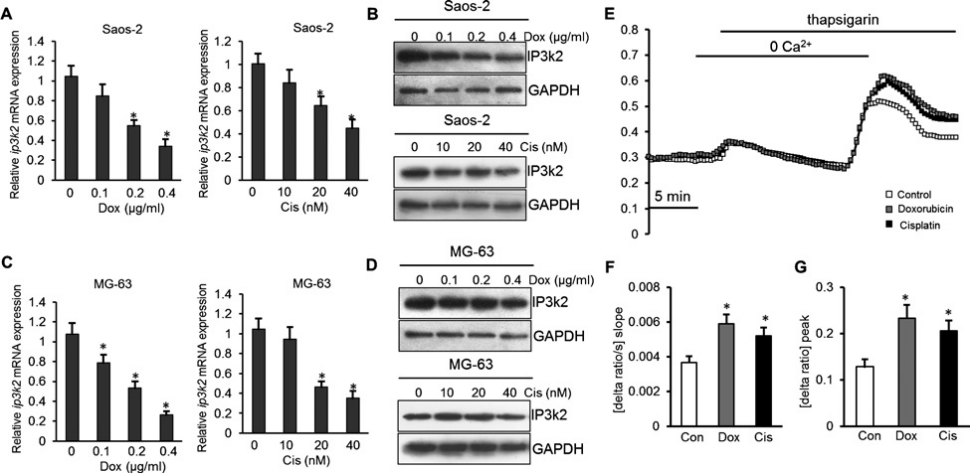
IP3K2 expression and Ca^2+^ entry were up-regulated in osteosarcoma cells following treatment with chemotherapeutic drugs (**A** and **B**) Analyses of IP3K2 mRNA and protein levels in Soas-2 cells after drug treatment. (**C** and **D**) Analyses of IP3K2 mRNA and protein levels in MG-63 cells after drug treatment. (**E**) Representative tracings of Fura-2 fluorescence ratio in fluorescence spectrometry during and after Ca^2+^ depletion with subsequent thapsigargin (1 μM) addition in Soas-2 cells without (white squares) and with presence of Dox (0.2 μg/ml, black squares) or Cis (20 nM, grey squares). (**F** and **G**) Arithmetic means (±S.E.M., *n*=5–6, each experiment 10–30 cells) of slope (**C**) and peak (**D**) increase in Fura-2 fluorescence ratio following Ca^2+^ readdition in the absence (white bars) and presence of drug (black or grey bars). * (*P*<0.05) indicate statistically significant difference from control.

Next, we tested whether the predicted target of *miR-140-5p* was down-regulated in response to drug treatment. The result of real-time PCR showed that the expression level of *ip3k2* mRNA was significantly lower after Dox and Cis treatment in Soas-2 and MG-63 cells (*P*<0.05, Figures 5A and 5C). And Western blot analysis revealed that IP3K2 protein levels were significantly lower in the drug treatment group than those control cells (Figures 5B and 5D). Furthermore, Fura-2 fluorescence was employed in order to test whether the differences in IP3K2 protein abundance were paralleled by corresponding differences in SOCE. As illustrated in Figure 5, both peak and slope of SOCE were significantly higher after Dox (0.2 μg/ml) and Cis (20 nM) pretreatment than in control cells. The Dox pretreatment increased the peak Ca^2+^ increase from 0.13±0.016 arbitrary units (*n*=6) to 0.23±0.028 arbitrary units (*n*=5) in Saos-2 cells. The Cis pretreatment increased the peak Ca^2+^ increase from 0.13±0.016 arbitrary units (*n*=6) to 0.21±0.023 arbitrary units (*n*=5) (Figures 5E–5G). These results showed that the decreased expression of IP3K2 was paralleled by corresponding differences in SOCE in response to drug treatment.

### Inhibiting IP3k2 protein expression promotes autophagy in response to anticancer drug treatment

**Figure 6:**
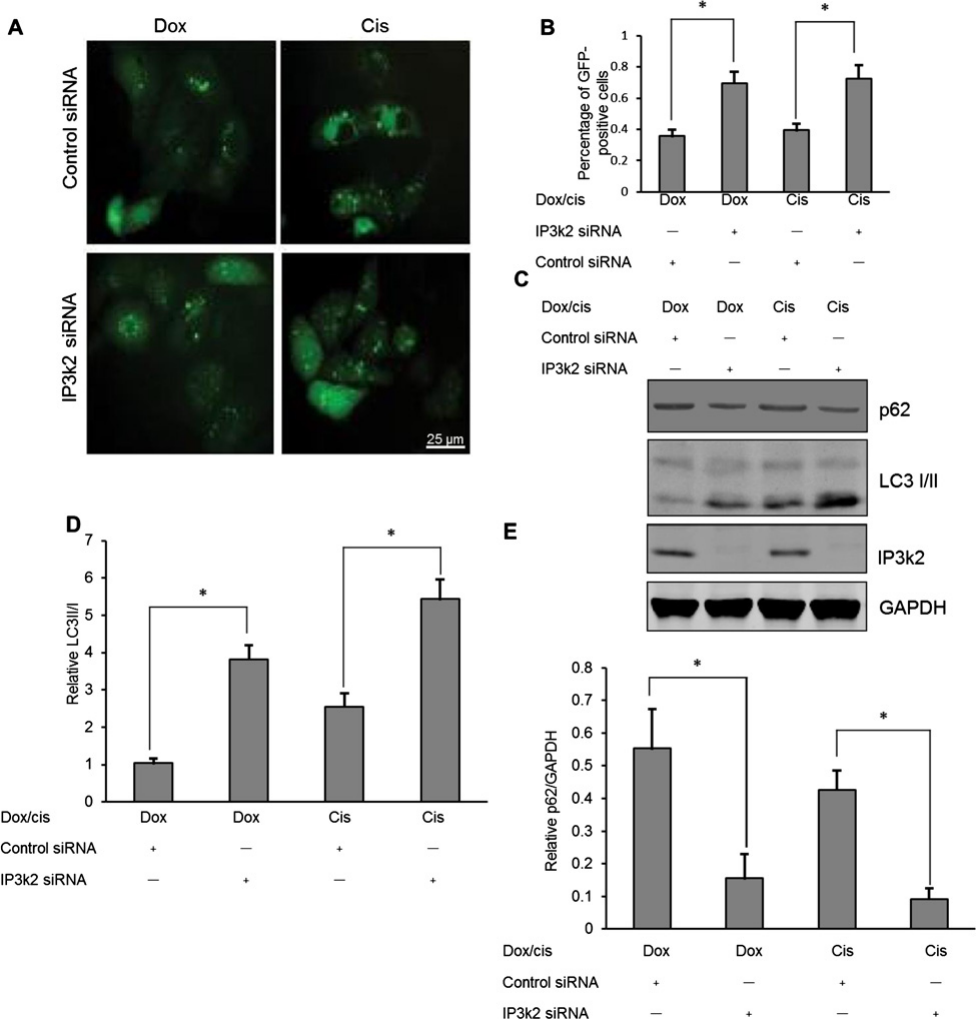
IP3k2 regulates autophagy in anticancer drug treatment (**A**) LC3 punctas under fluorescence microscopy in Saos-2 cells following 0.1 μg/ml Dox or 10 μM Cis treatment and IP3k2 siRNA or control siRNA transfection. (**B**) Quantitative analysis of the punctate GFP-LC3 dots. (**C**) Western blotting of LC3-I/II and p62 in IP3k2 siRNA or control siRNA-transfected Saos-2 cells. (**D**) Relative expression of LC3-II to LC3-I in IP3k2 siRNA or control siRNA-transfected Saos-2 cells. (**E**) Relative expression of p62 to GAPDH in IP3k2 siRNA or control siRNA-transfected Saos-2 cells. Independent experiments were performed in triplicate. *P<0.05 compared with control.

To determine the possible contribution of IP3k2 protein to autophagy in drug-treated osteosarcoma cells, IP3k2 protein expression was inhibited in Saos-2 cells via transfection with IP3k2 siRNA or control siRNA. The effect of inhibiting was determined by western blot shown in Figure 6(C). The level of autophagy was determined in Saos-2 cells following IP3k2 siRNA transfection. There were more GFP-positive dots (LC3 punctas) in the Saos-2 cells that had been transfected with IP3k2 siRNA when compared with transfection with control siRNA (Figures 6A and 6B, *P*<0.05). In addition, significantly higher conversion levels of LC3-I to LC3-II and decreased expression levels of p62 were also confirmed in the osteosarcoma cells transfected with IP3k2 siRNA (*P*<0.05, Figures 6C–6E). These results confirm that overexpression of IP3k2 contributes to anticancer drug-induced autophagy in osteosarcoma cells.

## DISCUSSION

The molecular mechanism of the sensitivity or resistance of cancers to chemotherapy is complex, involving multiple processes such as drug transport, drug metabolism, DNA repair, apoptosis and autophagy. Traditionally, DNA, mRNA and proteins have been most focused on as the targets and modulators of therapy. Therefore, mutations, copy number changes and epigenetic variables at the DNA level and expression changes at the mRNA and protein levels have been widely studied to probe mechanisms that determine the pharmacologic response [39–41]. Up-regulated autophagy has been found in various cancer cells facing therapeutic stress and contributes to the chemotherapy resistance [42,43]. Autophagy blocking in cancer cells is emerging as a novel approach to enhance the sensitivity of chemotherapy in cancers [44,45].

Tight control of autophagy is essential for normal or tumour cells to survive, and recent advances in this field have begun to unveil the molecular mechanisms underlying autophagy regulation [46]. A connection between inositol-1,4,5-triphosphate (IP3) and autophagosome formation has been also proposed [47,48]. IP3 is fundamental for Ca^2+^ homoeostasis since coupling its receptor (IP3R) functions as an actual gateway for every Ca^2+^ pulse originated from the ER [49]. Increases in [Ca^2+^]_c_ mediate autophagy in mammalian cells [50] and compounds acting via this pathway (e.g. vitamin D, ATP and ionomycin) are able to promote it quite efficiently. Ca^2+^-mediated autophagy seems to principally occur via Ca^2+^/calmodulin-dependent kinase-β (CaMKKβ)-dependent activation of AMPK thus leading to an efficient inhibition of mTORC1. Increases in the [Ca^2+^]_c_ can also activate death associated protein kinases and calpain proteases (both Ca^2+^-dependent enzymes) that have been linked to the regulation of the autophagic process [51]. In this study, we found that inhibiting IP3k2 up-regulates anticancer drug-induced but not basal level of autophagy, to promote tumour cell survival in exposure to anticancer drugs in osteosarcoma cancers. To the best of our knowledge, this is the first time to implicate the IP3-kinase is involved in control of autophagy in osteosarcoma cells.

Notably, miRNAs can regulate a multitude of targets and biological networks in autophagy [52,53]. A previous study indicated clear roles of miRNAs in autophagy induction, autophagic vesicle nucleation, autophagic vesicle elongation and vesicle fusion to lysosomes [52]. The present study confirmed that during treatment with Dox or Cis in osteosarcoma cells, *miR-140-5p* expression was strongly induced. The increased *miR-140-5p* expression facilitated tumour cell proliferation via up-regulating autophagy, thus, facilitated the resistance of osteosarcoma cells to Dox or Cis. In conclusion, the present study has demonstrated that anticancer drug treatment up-regulates *miR-140-5p* expression in osteosarcoma cells. Overexpression of *miR-140-5p* induces the activation of autophagy, which promotes tumour cell survival and chemoresistance. These observations reveal a novel role for *miR-140-5p* in chemotherapy resistance during the treatment of osteosarcoma.

The role of autophagy in the tumour cell's sensitivity or resistance to chemotherapy is complex. In osteosarcoma, as shown in other tumours, autophagy plays a dual role either by promoting cell survival and tumour cell resistance to chemotherapy or by acting as one of the mechanisms responsible for chemotherapy-induced cell death. Better understanding of the molecular pathways that govern the process of autophagy will allow identification of a mode to modulate these pathways in order to enhance the activity of chemotherapy. The present study has demonstrated that anticancer drug treatment up-regulates *miR-140-5p* expression in osteosarcoma cells. Overexpression of *miR-140-5p* induces the activation of autophagy, which promotes tumour cell survival and chemoresistance. These observations reveal a novel role for *miR-140-5p* in chemotherapy resistance during the treatment of osteosarcoma.

In summary, our study shows that *miR-140-5p* targeted IP3k2 and inhibited the IP3k2-mediated autophagy in osteosarcoma cells during the chemotherapy, and sensitized the osteosarcoma cells to anticancer drugs by inhibiting cell proliferation. These findings identified the novel tumour stimulative role of *miR-140-5p* in IP3k2-mediated autophagic chemotherapy resistance during the treatment of osteosarcoma.

## Abbreviations

CCK-8: Cell Counting Kit-8
Cis: cisplatin
Dox: doxorubicin
IP3k2: inositol 1,4,5-trisphosphate kinase 2
MTOR: mechanistic target of rapamycin
PtdIns3K: class III phosphatidylinositol 3-kinase
qPCR: quantitative PCR
SOCE: store-operated Ca^2+^ entry
